# Maternal Oxytocin Is Linked to Close Mother-Infant Proximity in Grey Seals (*Halichoerus grypus*)

**DOI:** 10.1371/journal.pone.0144577

**Published:** 2015-12-23

**Authors:** Kelly J. Robinson, Sean D. Twiss, Neil Hazon, Patrick P. Pomeroy

**Affiliations:** 1 Sea Mammal Research Unit, Scottish Oceans Institute, University of St Andrews, St Andrews, Fife, United Kingdom; 2 School of Biological and Biomedical Sciences, Durham University, South Road, Durham, United Kingdom; 3 Scottish Oceans Institute, University of St Andrews, Scotland, United Kingdom; University of Missouri, UNITED STATES

## Abstract

Maternal behaviour is a crucial component of reproduction in all mammals; however the quality of care that mothers give to infants can vary greatly. It is vital to document variation in maternal behaviour caused by the physiological processes controlling its expression. This underlying physiology should be conserved throughout reproductive events and should be replicated across all individuals of a species; therefore, any correlates to maternal care quality may be present across many individuals or contexts. Oxytocin modulates the initiation and expression of maternal behaviour in mammals; therefore we tested whether maternal plasma oxytocin concentrations correlated to key maternal behaviours in wild grey seals (*Halichoerus grypus*). Plasma oxytocin concentrations in non-breeding individuals (4.3 ±0.5 pg/ml) were significantly lower than those in mothers with dependent pups in both early (8.2 ±0.8 pg/ml) and late (6.9 ±0.7 pg/ml) lactation. Maternal plasma oxytocin concentrations were not correlated to the amount of nursing prior to sampling, or a mother’s nursing intensity throughout the dependant period. Mothers with high plasma oxytocin concentrations stayed closer to their pups, reducing the likelihood of mother-pup separation during lactation which is credited with causing starvation, the largest cause of pup mortality in grey seals. This is the first study to link endogenous oxytocin concentrations in wild mammalian mothers with any type of maternal behaviour. Oxytocin’s structure and function is widely conserved across mammalian mothers, including humans. Defining the impact the oxytocin system has on maternal behaviour highlights relationships that may occur across many individuals or species, and such behaviours heavily influence infant development and an individual’s lifetime reproductive success.

## Introduction

Infant survival in typical mammalian species depends crucially on appropriate maternal behaviour, requiring substantial investment of a mother’s time and energy to be successful [1]. Some individuals fail to rear offspring successfully, or rear offspring inefficiently in energetic terms [2–6] which can impair infant development [7]. Individual maternal behaviour can vary [8] according to the age of the individual [9], their habitat [10] and the mother’s nutritional status during the dependent period [11]. The physiological processes controlling maternal behaviour should be conserved throughout reproductive events and should be replicated across all individuals of a species, as raising offspring successfully is subject to strong selection pressure [12].

Oxytocin is a neuropeptide hormone that modulates maternal [13] and social behaviour [14, 15], promotes parturition and lactation in mammals, and has been connected to maternal behaviour expression [16–19] and bond formation with dependent infants [20–22]. Oxytocin is a good candidate for the investigation of widespread physiological regulators of maternal behaviour as it is highly conserved in both structure and function across many mammalian species and other vertebrate clades [23].

Many studies on humans and captive or domestic animal species show direct links between central and peripheral oxytocin concentrations and maternal behaviour. High plasma oxytocin concentrations correlate to optimal maternal behaviour in humans (*Homo sapiens* [24]), to mothers checking, affectionately touching and talking to their babies (humans [18]) and to high frequencies of nursing and grooming (rhesus macaque, *Macaca mulatta* [25]). Peripheral injections of oxytocin additionally increase maternal aggression towards foreign lambs in sheep (*Ovis aries* [16]), increase pup retrieval in female voles (*Microtus ochrogaster* [26]) and increased the time spent with pups in meerkats (*Suricata suricatta* [27]). However no studies have examined whether endogenous oxytocin concentrations in wild mammalian mothers are linked to any aspect of their maternal behaviour. Investigating oxytocin in the wild is crucial, as captive conditions rarely represent all aspects of natural systems and conditions can impact significantly on physiological parameters of study animals (reviewed in [28]). Captive mothers receive veterinary care and appropriate nutrition, often without foraging costs, thus material resources and time to interact with offspring are increased compared to wild counterparts [29]. It would be crucial to our understanding of maternal physiology to determine if correlations between oxytocin and types of maternal behaviour observed in laboratories, farms or zoos actually exist in natural populations.

The repertoire of maternal behaviour of a north Atlantic phocid, the grey seal (*Halichoerus grypus*), provides an excellent model to investigate physiological constraints on maternal behaviour expression in a natural population. Phocid seals have a comparable plasma clearance rate and basal concentrations of plasma oxytocin compared to humans and rodent model species [30]. Mother-pup pairs may be observed ashore during the 18 day dependent period [31] and temporally stable, individually distinctive pelage markings allow mothers to be identified repeatedly [32]. Additionally, the breeding system for grey seals is both spatially and temporally contained, enabling the measurement of factors affecting maternal success and the outcome of single reproductive episodes. Mothers each have one pup and fast while producing milk with one of the highest fat contents of all mammals (60% [33]). Their lactation period and opportunity to invest in their offspring is extremely brief for a mammalian species [33] and the pup’s mass at weaning correlates with its survival probability [34]. Furthermore, a mother’s future reproductive success is impaired by excessive breeding effort [31]. While the majority of pups born are weaned successfully, maladaptive behaviours such as abandonment do occur [35]. Fostering [3] and allosuckling [36] also remain relatively common although there is little evidence of kin clustering on grey seal colonies [37]. There is no satisfactory explanation for why mothers invest in non-filial pups or display behaviours that almost inevitably lead to the death of their pups. Oxytocin concentrations in grey seal mothers may provide a physiological mechanism explaining this individual variation in pup rearing behaviour.

Two methodologies can be used to link oxytocin concentrations from samples of peripheral substrates to types of behaviour. Samples can be collected directly after specific natural behaviours or experimental treatments to provoke behaviour to infer a relationship between the behaviour’s expression and oxytocin release (e.g. [38, 39]). Alternatively, basal plasma oxytocin concentrations can be collected and analysed across individuals showing different behavioural repertoires, a method used previously to link patterns of peripheral oxytocin concentrations to a variety of psychological or developmental disorders in humans [40–43]. Basal plasma oxytocin concentration during pregnancy in humans was a strong predictor of maternal bonding post-partum [44]. Therefore, basal plasma oxytocin concentration is a strong candidate for quantifying the mother-infant bond and the subsequent maternal behaviour she displays towards it, manifested through interaction and proximity. While using peripheral measurements of oxytocin over measurements from central brain regions has been questioned [45], coordinated release of central and peripheral oxytocin occurs during a variety of aspects of maternal behaviour [46]. The difficulty of collecting cerebrospinal fluid from wild, free-ranging individuals makes the use of plasma preferable outside captive or laboratory environments.

We predict that mothers with dependent pups will have higher basal concentrations of plasma oxytocin compared to non-breeding individuals, and that maximal oxytocin concentrations occur during or immediately after nursing bouts. We will investigate whether any variation in basal maternal oxytocin concentrations is correlated to the amount of time mothers spend nursing in the days prior to sampling. Finally we hypothesise that maternal basal oxytocin concentrations vary significantly between individuals and this correlates with their subsequent maternal behaviour.

## Materials and Methods

### Ethical Statement

All applicable international, national and/or institutional guidelines for the care and use of animals were followed in this study. All animal procedures were performed under the UK Home Office project licence #60/4009 and conformed to the UK Animals (Scientific Procedures) Act, 1986. All research received prior ethical approval from the University of St Andrews Animal Welfare and Ethics Committee and the School of Biology’s Ethics Committee. Highly trained and experienced personnel performed all capture and handling procedures of grey seal mothers and appropriate anaesthesia (Zoletil 100™) was used during all captures to minimise any stress the capture and sampling procedures may cause to individuals that participated in this study.

### Study sites and animals

Field work was conducted on the North Rona, Scotland (59°06’N, 05°50’W), a grey seal breeding colony with a long term research project monitoring it. This study ran from 3^rd^ October– 31^st^ October 2010 and 2^nd^ October– 2^nd^ November 2011. Scottish Natural Heritage (SNH) gave permission for fieldwork to be conducted on the Isle of May and Barvas Estates and SNH gave permission for fieldwork to be conducted on North Rona.

Plasma samples were collected from 31 mothers, and behavioural observations were taken from 21 mothers (Table 1). Five mothers with plasma samples and behavioural observations occurred in both study years, and ten mothers with plasma samples occurred in both study years. All mothers observed were known from the long-term dataset of returning breeding females for this colony and were not first time mothers [31]. Mothers were identified by unique identification markings (natural pelage patterns, or applied tags or brands [47]). Behavioural observations were restricted to mothers first seen either prepartum or with young (stage I [48]) pups.

**Table 1:** Number of mothers on North Rona included in this study across the two study years.

Study Year	Number of mothers with plasma samples	Number of mothers with behavioural observations
**2010**	18	12
**2011**	13	9

Plasma samples from non-breeding females were collected on the Isle of May (56°11’N, 02°33’W) colony in Scotland between 30^th^ October– 3^rd^ December 2011. Samples were collected from eight juvenile female grey seals between 12 and 24 months of age to ensure they were non-breeding individuals.

### Plasma Sampling and Analysis

All study mothers were captured twice during the lactation period to obtain plasma samples at 1–7 days after the pup’s birth (‘early lactation’) then 9–15 days after the first sampling event (‘late lactation’). Mothers were approached and administered 1ml/100kg of Zoletil 100™ (Vibrac) intramuscularly using a pressurised dart, then left for 10 minutes to allow the drug to take effect [31]. Mothers were restrained in a net for sampling. Pups were caught and restrained manually to ensure they did not become separated from their mothers during the procedure. Non-breeding individuals were approached and captured manually using a specially designed capture bag by an experienced investigator. Capture methodology (chemical immobilisation or physical restraint) have been shown to have no effect on plasma oxytocin concentration [30]. Blood was drawn into 10ml lithium heparin vacutainers from the extradural vein without addition of aprotinin and stored on ice until they could be spun and the plasma aliquoted and frozen at -20°C. This sample storage methodology produces negligible oxytocin degradation over at least a two year period [30].

Our capture techniques meant that there was always a 10 minute wait for the drug to immobilise a mother before a plasma sample could be collected. We therefore were able to document any pre-sampling nursing that may have altered basal plasma oxytocin concentrations, as mothers were observed prior to darting to ensure it was safe to proceed with the capture, and were observed during the entire 10 wait for the drug to take affect. This wait would eliminate any plasma oxytocin peaks triggered by pre-capture nursing as oxytocin has a short half life in plasma (between 3–4 minutes in non-pregnant human women [49]). There were two instances during the study where pups initiated nursing during immobilisation induction. In both cases, the nursing bout was allowed to finish naturally and was followed by immediate plasma sampling of the mother to minimise the oxytocin lost to natural clearance of the hormone from circulation. These two samples enabled us to document what plasma oxytocin concentrations occur in grey seals during nursing, but were not included in analysis with behavioural data.

Plasma was analysed for oxytocin using an ELISA (Enzyme-Linked Immunosorbent Assay, Assay Designs Inc, Ann Arbor, MI, USA) with each sample undergoing solid-phase extraction using Sep-Pak C18 columns prior to analysis following the methodology validated for detecting phocid plasma oxytocin [30]. Plates were read using a BioTek ELx800 reader. The standard curve and assay results for all plates were fitted using the calibFit package [50] in R version 2.9.2 [51]. Recovery rates for the extraction and ELISA procedure were 107.2% (n = 10), intra-assay coefficient of variance (COV) for this assay was 6.2% and inter-assay COV over the 12 plates used in this study was 12.3%.

### Behavioural observations

A total of 153 hours of observations were collected using the behavioural categories described in our ethogram (S1 Table, adapted from [52–56]). During each day of observations, two mother-pup pairs were observed over nine consecutive hours every day using scans every 30 seconds for 10 minutes, followed by a five minute break to record other data from the colony, giving on average 782 (range: 528–880) scans per mum-pup pair per day. The distance from mother to pup (MPD) was estimated visually in adult body lengths (1 adult body length ≈ 2 metres) every 15 minutes. No observations were taken on capture days. Each mother-pup pair had one day of behavioural observations taken after plasma samples where collected in early and/or late lactation.

Behaviour was classified into 24 mutually exclusive categories (S1 Table). Two of these categories were not included in subsequent analysis (‘out of sight’ and ‘comfort move’) as we assumed they would not be linked to hormonal concentrations, and eight categories were recorded infrequently (recorded in <3 days of the study) or are not associated with maternal care of offspring and were therefore eliminated from subsequent analysis (general behaviours: ‘other’, aggressive behaviours: ‘chase’, ‘flee’ and ‘vocalisation’, maternal behaviours: ‘birth’, sexual behaviours: ‘attempted copulation’, ‘failed copulation’ and ‘copulation’). Sexual behaviours were only observed in the days after late lactation plasma sampling, and only occurred in two of the observed days in the study. For analytical purposes the remaining 14 categories were combined into seven broader, biologically relevant behaviour groups (Table 2). Scan data were used in the general additive mixed models (GAMMs, see below) however, to enable comparisons to previous studies of maternal behaviour in grey seals, the daily percentage of time a mother was observed in each behavioural group was estimated by dividing the number of scans in which a mother showed a behaviour in the groups (Table 2) by the total numbers of scans for which she was observed for that day and multiplying by 100.

**Table 2:** The seven behaviour groups used for analysis with plasma oxytocin concentrations and the ethogram categories (S1 Table) included in them.

Behaviour group	Ethogram Categories (S1 Table) included
**Resting**	‘Rest’ and ‘Head up rest’
**Nursing**	‘Presenting’ and ‘Nursing’
**Interacting with pup**	‘Interacting with pup’ and ‘Flippering’
**Checking pup**	‘Check pup’
**Alert**	‘Alert’
**Maternal Aggression**	Open mouth threats to males and females, ‘Aggressive flippering’, ‘Lunge’ and ‘Bite’
**Locomotion**	‘Locomotion’

Of the 21 study mothers across the two years, seven had data only from either the early or late period of lactation. The remaining 14 mothers all had plasma samples and behaviour observations for both early and late lactation, giving a total of 35 sets of paired behaviour data and plasma oxytocin concentrations. Observations were targeted on recently captured individuals to make oxytocin concentrations detected on the capture day as relevant as possible. Behaviour was recorded the day after capture days in 18 sets; however as some study mothers were not always in sight, observations in the remaining cases varied from one to six days post-capture.

### Statistical analysis

All analyses were performed using the statistical package R 2.15.0 [51].

### Basal plasma oxytocin concentrations in grey seals

The plasma oxytocin concentrations from non-breeding female grey seals and mothers with pups required a natural logarithmic transformation as the original data were not normally distributed (Shapiro Wilks test: p<0.001). The transformed data were normally distributed with homogenous variance across all groups. A one way ANOVA with a Tukey honest significant differences *post-hoc* test was used to test for differences in plasma oxytocin concentrations between the two sampling opportunities for mothers with dependent pups (early and late lactation) and the non-breeding group. The two data points for mothers after a nursing bout were not included in the analysis due to the low sample size.

### Plasma oxytocin concentrations and nursing prior to capture

Basal plasma oxytocin concentrations and the percentage of observed time a mother spent nursing the day before sampling and one to nine days pre-capture were examined using Pearson’s correlation coefficient. Grey seals typically nurse their pups every four hours [33], enabling us to document nursing behaviour regularly throughout observation days. A total of 27 days of observations prior to plasma sampling were collected. Where possible, observations were collected the day before either an early or late capture occurred (9 sets of observation data) but when this was not possible, observations were also collected from study mothers up to nine days before a capture day (18 sets of observation data). The percentage of time a mother was observed nursing was estimated by dividing the number of scans in which a mother was recorded nursing by the total numbers of scans for which she was observed for that day and multiplying by 100.

Additionally, basal plasma oxytocin concentrations at late lactation and the nursing intensity a mother showed during the 18 day dependant period were examined using Pearson’s correlation coefficient. The ‘nursing intensity’ of a mother was calculated by taking the mean of the percentage of observed time per day a mother spent nursing over the 18 day dependant period. This was done to establish whether mothers that typically spent more time nursing in a day had higher basal oxytocin concentrations than mothers that spent less time nursing, due to more frequent elevations of plasma oxytocin from lactation. All mothers in dataset with a ‘late lactation’ plasma sample and behavioural data collected over the 18 day dependant period (n = 15) were used for this correlation, and all mothers had between three and six days of observation data across the 18 day dependant period to obtain a mean from.

### Plasma oxytocin concentrations and subsequent maternal behaviour

GAMMs [57] were used to analyse variables affecting MPD or the frequency of the seven maternal behaviour groups (Table 2), resulting in eight models. Biologically plausible explanatory variables used in these models were plasma oxytocin concentration for mothers, the number of days that had elapsed since sampling, the date of the pup’s birth, sample timing during the season (early or late lactation) and the pup’s sex. Date of pup birth, pup sex and time of behavioural sampling in lactation have been shown to influence the behaviour exhibited by individuals on a breeding colony [31, 58, 59]. Models were fitted using the multiple generalized cross validation library mgcv [60]. The identities of mothers were fitted as a random effects smooth [61] to control for pseudo-replication in the dataset from using some of the same individuals over the two years of the study and to control for consistent individual differences in behaviour [56]. The smoothing parameters were set by maximum likelihood to reduce the risk of overfitting associated with other methods [62]. The MPD model was fitted with a Gamma error distribution. The models constructed for each of the seven behavioural groupings were fitted with binomial distributions because the scan data were transformed into the proportion of time individuals were observed performing and not performing behaviours listed in the groupings. Model selection was done by backwards stepwise elimination through examination of R^2^ values, AIC values, QQ and residual plots to identify the best model for the data.

Finally, basal plasma oxytocin concentrations at early lactation and the nursing intensity a mother showed during the 18 day dependant period were examined using Pearson’s correlation coefficient. The ‘nursing intensity’ of a mother was calculated as described above. This was done to establish whether oxytocin concentrations early in the lactation period drive how intensely a mother nurses her pup. All mothers in dataset with an ‘early lactation’ plasma sample and behavioural data collected over the 18 day dependant period (n = 14) were used for this correlation, and all mothers had between three and six days of observation data across the 18 day dependant period to obtain a mean from.

### Data Availability

All relevant data are within the paper and its Supporting Information files (S4 Table).

## Results

### Basal plasma oxytocin concentrations in grey seals

**Fig 1:**
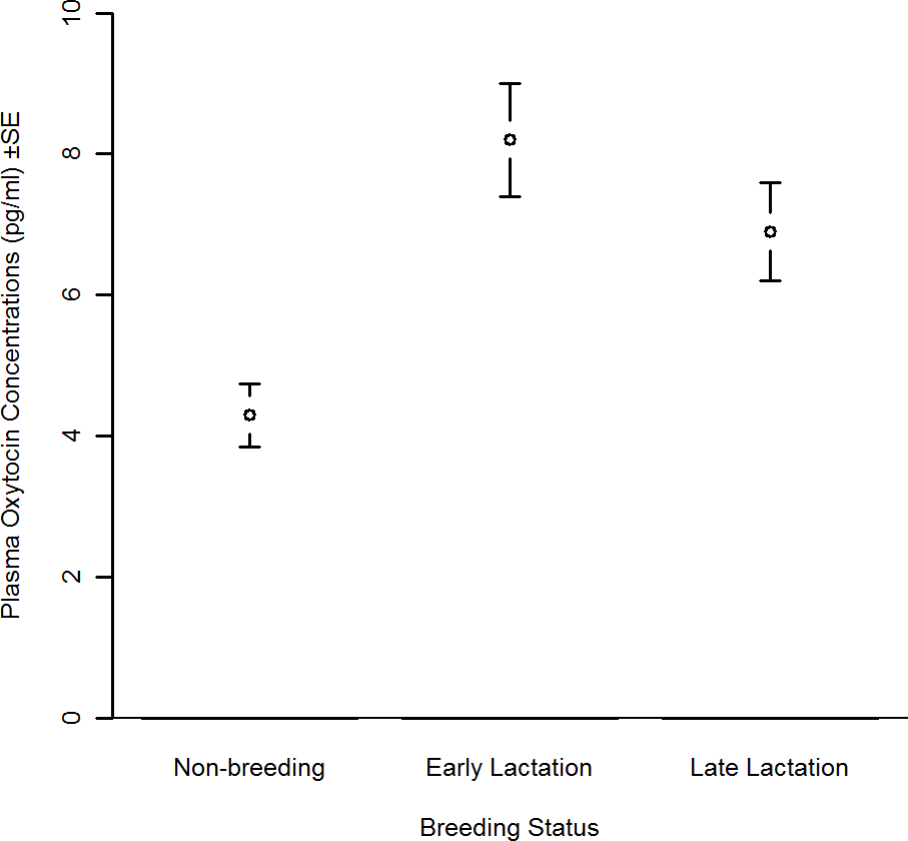
Basal Plasma Oxytocin and Breeding Status. Mean basal plasma oxytocin (pg/ml) across three different breeding statuses, non-breeding females (n = 8), mothers with dependent pups in early lactation (n = 31) and mothers with dependent pups in late lactation (n = 31) with standard error bars.

Basal plasma oxytocin concentrations differed by breeding state, specifically, non-breeding individuals had significantly lower plasma oxytocin concentrations (4.3 ±0.5pg/ml) (ANOVA: F_2,67_ = 8.3, p<0.001) than mothers with dependent pups in both early lactation (Tukey honest significant difference test, p<0.001) or late lactation (Tukey honest significant difference test, p = 0.01) (Fig 1). No significant differences were detected between mothers in early and late lactation (mean ±SE: 8.2 ±0.8 pg/ml and 6.9 ±0.7 pg/ml respectively, Tukey honest significant difference test, p = 0.23).

### Plasma oxytocin concentrations during nursing in grey seals

The two actively nursing mothers (not included in the statistical comparison) had concentrations of 30.5pg/ml and 39.8pg/ml, up to five times higher than concentrations in mothers with pups who were not nursing.

### Plasma oxytocin concentrations and nursing prior to capture

Prior to sampling, mothers spent 3.1% (±1.1%) of the day before capture nursing their pups and 3.9% (±0.5%) of their time nursing their pups on a single day within 1–9 days before capture. This is comparable to the proportion of time mothers spend nursing pups in previous studies of Scottish grey seals (S2 Table). There was no significant correlation between plasma oxytocin concentrations and the time a mother spent nursing the day before sampling (r = 0.089, p = 0.8) or on a single day within 1–9 days prior to sampling (r = 0.018, p = 0.9).

The nursing intensity different mothers exhibited across the 18 day dependant period varied greatly, with some mothers spending, on average, three times the amount of time in a day nursing their pups (lowest mean: 2.4% ±0.4%, highest mean: 9.1% ±2.3%, both from four days of observations). However this variation was not correlated with a mother’s plasma oxytocin concentration at late lactation (r = 0.28, p = 0.3). Therefore, more frequent elevation of basal oxytocin concentrations due to more frequent lactation events does not cause a rise in basal plasma oxytocin concentrations across the dependant period.

### Plasma oxytocin concentrations and subsequent maternal behaviour

After sampling, the mean percentages of time that mothers spent in the seven behavioural groups were; 80.9% (±0.9%) resting, 5.04% (±0.5%) nursing, 1.3% (±0.2%) interacting with their pup, 3.5% (±0.3%) checking their pup, 3.6% (±0.3%) alert to their surroundings or other seals, 0.7% (±0.1%) in maternal aggression and 1.2% (±0.2%) in locomotive behaviours. The mean percentage of time mothers spend in each behavioural group in early and late lactation are reported in Table 3. These figures are comparable to the proportion of time mothers spend in these activities in previous studies of Scottish grey seals (S2 Table).

**Table 3:** The seven behaviour groups for grey seal mothers and mean percentage time recorded per day for each behaviour group in early and late lactation (±SEM).

Behaviour group	Mean percentage time spent per day (±SEM)	
	Early Lactation	Late Lactation
**Resting**	82.9% (±0.9)	78% (±1.4)
**Nursing**	3.8% (±0.5)	6.7% (±0.8)
**Interacting with pup**	0.9% (±0.3)	1.9% (±0.4)
**Checking pup**	3.3% (±0.4)	3.8% (±0.5)
**Alert**	3.6% (±0.4)	3.7% (±0.4)
**Maternal Aggression**	0.7% (±0.2)	0.8% (±0.2)
**Locomotion**	1.6% (±0.2)	0.7% (±0.2)

Across the eight GAMMs modelling maternal behaviour throughout lactation, plasma oxytocin concentration was a significant explanatory variable in two of the models. The six models where plasma oxytocin concentrations were not a significant explanatory variable for maternal behaviour are reported in the supporting materials (S3 Table).

**Fig 2:**
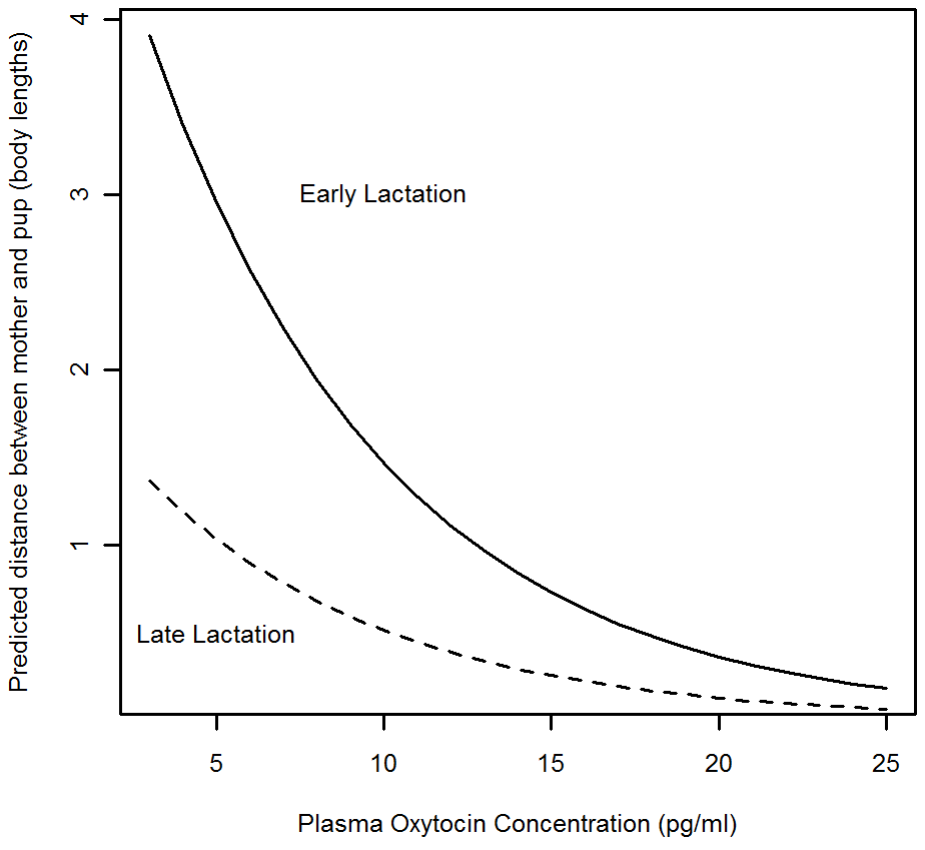
Maternal Plasma Oxytocin and Infant Proximity. GAMM output of the predicted relationship between mother-pup distance (MPD, body lengths) and mother’s plasma oxytocin concentration (pg/ml) in early (solid line) and late (dashed line) lactation.

The mean distance between a mother and her pup (MPD) was negatively correlated with plasma oxytocin concentrations (p = 0.006). Mothers during late lactation had lower MPDs than mothers in early lactation (p = 0.004) (Fig 2, Table 4). The number of days observations were recorded since plasma sampling, the pup’s birth date and sex were not significant explanatory variables and were eliminated from the final model during the selection process. Mothers’ identity was not a significant explanatory variable in the model; however it was retained to improve the model fit.

**Table 4:** Significant fixed effect variables from the only GAMMs for maternal behaviour where plasma oxytocin concentrations significantly impacted on the dependent variable, their estimates, standard errors and p values.

Model Number: Dependent variable	Explanatory variable	Estimate	Standard Error	P value
**Model 1: MPD**	Maternal plasma oxytocin concentration (pg/ml)	-0.14	0.047	**0.0058**
	Sample timing during the season (early/late)	-1.05	0.32	**0.0036**
	Smooth term for mother’s identity	-	-	0.85
**Model 2: Maternal Aggressive behaviour**	Maternal plasma oxytocin concentration (pg/ml)	0.071	0.03	**0.0094**
	Sample timing during the season (early/late)	0.63	0.24	**0.011**
	Pup sex (male/female)	5.9	3.9	0.13
	Pup birth date	0.39	0.18	**0.028**
	Number of days behaviour was recorded after sampling	0.06	0.11	0.59
	Smooth term for mother’s identity	-	-	**<0.001**

The nursing intensity different mothers exhibited across the 18 day dependant period was not correlated with a mother’s plasma oxytocin concentration at early lactation (r = 0.12, p = 0.7). Therefore, concentrations of oxytocin early in the lactation are not driving subsequent nursing intensities observed over the dependant period.

The amount of maternal aggression displayed was significantly positively correlated with her plasma oxytocin concentration (p = 0.009). Additionally, the identity of the mother caused significant variation in the amount of aggressive behaviour she displayed (p<0.001). Mothers during late lactation were more aggressive than mothers in early lactation (p = 0.01) and mothers that gave birth later in the breeding season were more aggressive than those giving birth early in the season (p = 0.03) (Table 4). The number of days observations were recorded since plasma sampling and the pup’s sex were not significant explanatory variables in the model, however they were retained to improve the model fit. While this model indicates that plasma oxytocin concentrations have a significant impact on the amount maternal aggression, the actual effect size is so small (causing a 0.005% change in aggression exhibited across 0-25pg/ml plasma oxytocin) that it would in reality have no biologically important effect on the amount of aggressive behaviour a mother displayed. As stated above, mothers spend a mean of 0.7% of their time performing maternal aggression behaviours, which equates to approximately three minutes of one day of observations in this study. Therefore even at the highest basal oxytocin concentrations, mothers would only spend 0.009 of a second per day performing more maternal aggression behaviours compared to mothers with the lowest basal oxytocin.

## Discussion

This is first study to link any expression of maternal behaviour to endogenous oxytocin concentrations in a wild population. Our results show that basal plasma oxytocin concentrations elevate significantly post-partum while a mother is nurturing her dependent pup, and a mother’s plasma oxytocin concentration is correlated to a key maternal behaviour which impacts on infant survival. Our findings are consistent with those from studies on domesticated [22], artificially maintained [25] or artificially manipulated animals [27] which demonstrate that peripheral oxytocin concentrations are linked to a variety of maternal behaviours.

### Plasma oxytocin concentrations are elevated when individuals have dependent offspring

Grey seal mothers have significantly higher plasma oxytocin concentrations than non-breeding individuals in early and late lactation. Mothers sampled immediately after nursing, as expected, had the highest plasma oxytocin concentrations detected in our study. The pattern of oxytocin concentrations across reproductive state in grey seals is consistent with that detected respectively in non-breeding adult humans (1.8pg/ml in [63], 1.7pg/ml in [64]), human mothers with dependent babies (10.8pg/ml in [65], 5.4pg/ml in [66]) and human mothers while nursing (53.2-224pg/ml in [65], 13-54pg/ml in [66]). The present data provide further evidence that the oxytocin system in grey seals is comparable to that found in humans, and that findings in phocid species may be applicable to other mammalian species [30].

Although elevated plasma oxytocin concentrations seen in mothers could be a by-product of the frequent plasma oxytocin peaks caused by nursing, our study found no correlations between plasma oxytocin concentrations and the time mothers spent nursing before sampling. We also found no evidence that mothers that show higher nursing intensity over the dependant period have higher basal oxytocin concentration. Nursing induced plasma oxytocin peaks immediately prior to sampling were also ruled out as a confounding factor in our analysis as our capture methodology chemically immobilises mothers prior to sampling, with a ten minute wait for the drug to take effect. During this time, any nursing could be observed and recorded, and any plasma oxytocin peaks from nursing prior to immobilisation would be rapidly cleared from circulation during this time period [67]. Thus any samples which could potentially contain elevated oxytocin concentrations from behaviours immediately prior to sampling, which would not represent the basal concentration for the mother, could be identified and excluded from the analysis. The variation in maternal oxytocin concentrations seen in our study mothers must therefore be due to factors other than nursing.

We propose that elevated plasma oxytocin concentrations are an indication of a functional mother-pup bond in our study mothers. Human mothers with high plasma oxytocin concentrations have strong mother-infant bonds [18, 44], are less likely to develop conditions symptomatic of a poor mother-infant bond such as postpartum depression [68] and in mother-infant dyads with a strong bond, there is greater peripheral oxytocin release from interactions with the offspring or infant stimuli [19]. Therefore, if a mother forms a bond with her offspring successfully, interacting with it or receiving stimuli from it causes peripheral oxytocin release, thus causing a transition from the basal concentrations observed in non-breeding individuals to those observed in breeding mothers.

If maternal bond presence or strength is the explanation of high basal plasma oxytocin concentrations in mothers, then a mother with a poor bond with her infant should have a low plasma oxytocin concentration. Opportunistic data from our study supports this. One mother’s plasma oxytocin concentration of 3.3pg/ml in early lactation in 2011 was comparable to concentrations seen in non-breeding individuals (4.5 ±0.5pg/ml), and was the lowest concentration from all of the study mothers, potentially indicating that she had a poor bond with her pup. This mother abandoned her pup on the fourth day post-partum in 2011, despite nursing and interacting with the pup at comparable levels to successful mothers prior to abandoning it. This mother had been in the long term study of the colony since 1996 and had successfully raised at least ten pups to weaning prior to this study and then subsequently in 2012 and 2013. The abandonment was also not due to the pup failing to thrive or being too sick or weak to respond to its mother, as after abandonment the pup successfully approached other mothers to suckle from in order to survive and was eventually adopted and raised successfully to weaning by a non-filial mother. Therefore, this individual was an experienced mother capable of exhibiting functional maternal behaviour and had a healthy, robust pup. While she did not have detailed behavioural data collected in both years of this study, we did obtain plasma samples from her in both years. In 2010 her early lactation plasma oxytocin concentration (9.9pg/ml) was three times higher than in 2011, and in 2010 she raised her pup to weaning successfully. This comparison exemplifies the contrast between the behaviour a mother exhibits when she has a high plasma oxytocin concentration compared to one similar to non-breeding individuals. Therefore we suggest that plasma oxytocin concentrations in mothers with dependent offspring are an indicator of the presence of a functional bond with their offspring and a mother with concentrations not exceeding non-reproducing levels may exhibit poor maternal behaviour, jeopardizing infant welfare.

The neurobiological mechanism for mother-infant bonding offers a potential explanation how such a crucial process can be disrupted resulting in poor mother-offspring bonds, and why this leads to low plasma oxytocin in mothers. Species with olfactory based offspring recognition systems (e.g. sheep), have a limited period of heightened sensitivity in the olfactory bulb of 1–4 hours after parturition, modulated by the release of neurotransmitters crucial to olfactory memory to allow mother-offspring bonds to form [20, 69]. If the bond is not established within this window, then there is no other opportunity in that breeding event to rectify this error [69]. These events can ultimately lead to abandonment, as human mothers with insecure mother-infant bonds and low plasma oxytocin have been found to react to stimuli associated with their infants with fear and disgust [19]. There is behavioural evidence indicating the importance of olfaction and the hour immediately after birth for mother-pup recognition in grey seal mothers, and this is indicative of a critical bonding period [35, 70]. There are many distractions that may prevent a grey seal mother from staying in close contact with her pup on a breeding colony; neighbouring breeding adults may attack the pair [35] and gull species harass mother-pup pairs to scavenge the placenta [70]. Such stochastic distractions may hinder or prevent bonding with the pup. However, such an event in one season has no impact on a mother’s ability to bond with her pup in successive seasons, nor does a string of successful breeding years predispose a mother to avoid this problem.

### Maternal plasma oxytocin concentrations and aggression

Our analysis showed that plasma oxytocin concentrations influenced the amount of aggression mothers directed towards other seals on the colony. However the effect size of oxytocin’s impact on aggressive behaviour was so small that the result has to be discounted, despite being consistent with manipulation studies that showed oxytocin injections increase maternal aggression outside the mother-infant dyad [16]. It is possible that plasma oxytocin concentrations are not accurately reflecting central concentrations of the hormone in relation to maternal aggression, and measurement of substrates such as cerebral spinal fluid (CSF) may show a larger, and biologically relevant, effect size.

### Mothers with high plasma oxytocin stay close to their infants

Plasma oxytocin concentration has a large effect on MPD, especially in early lactation, with mothers with higher concentrations of plasma oxytocin spending more of their time in close proximity to their pups. Artificially manipulating plasma oxytocin concentrations via intramuscular injections increased the time individuals spent with pups in meerkats [27] and it has been suggested that oxytocin promotes proximity seeking behaviours in humans [71]. However, this is the first time that proximity seeking behaviour, considered to be a key component of mammalian maternal behaviour [18] has been linked to endogenous oxytocin concentrations in mothers with dependent offspring. At this stage causal relationships between MPD and plasma oxytocin concentrations cannot be inferred directly, as exposure to infant stimuli, which would occur when in close proximity to an infant, has been shown to stimulate peripheral oxytocin release [17, 19]. Even if elevated oxytocin concentrations produce low MPD, oxytocin concentrations in central brain regions could be responsible for the variation in this behaviour, while peripheral oxytocin concentrations may simply reflect of the central concentrations of the hormone [72]. Whichever explanation is valid, it is clear that in seals, there is a relationship between high oxytocin concentrations and maintaining close proximity to dependent infants, a crucial behaviour for this species. Close maternal proximity reduces the likelihood of separation during lactation, which is credited for causing starvation, the most common cause of pup mortality on Scottish breeding colonies [73–75]. Therefore oxytocin is linked to a key maternal behaviour in seals, and any disruption to maternal bonding, which may impact on oxytocin concentrations as outlined above, could impact negatively on a pup’s welfare or survival. Additionally, if elevated oxytocin concentrations stimulate mothers to remain close to their pups, further oxytocin release could subsequently be triggered by stimuli from the infant [19], establishing a positive feedback loop in the mother. Oxytocin is linked to numerous reward and reinforcement pathways in the brain during optimal maternal behaviour expression [24, 76]. Oxytocin cells in the medial preoptic area (MPOA) of the hypothalamus are also specifically activated by pup stimuli in lactating female mice but not non-breeding individuals [77] and project to central brain regions while contributing to peripheral oxytocin release [78]. Oxytocin release from infant stimuli would therefore both trigger physiological reward pathways in the mother’s brain and continue to stimulate close proximity behaviour.

It is likely that pup growth and development throughout the lactation period explains the significance of lactation stage (early/late) in the relationship between mother-pup distance and a mother’s plasma oxytocin concentration. In early lactation, up to a week after birth, pups are small, weak and uncoordinated, so the distance between a mother and pup is mostly determined by the mother’s actions, which in turn can be impacted by her own internal physiological systems. However in late lactation, pups are larger, more mobile and capable of remaining with their mothers through their own actions. Therefore pups actively associating with their mothers in late lactation may explain why the correlation between mother-pup distance and plasma oxytocin concentration is not as strong as it is in early lactation.

Grey seal mothers have been shown to exhibit consistent individual differences (CIDs) in aspects of their maternal behaviour, specifically ‘Checking pup’ and ‘Alert’ behaviours [54–56]. Such CIDs may be indicative of differing behavioural types of maternal styles [56]. In this study, many of the behaviours observed, maternal or otherwise, varied significantly according to individual, with the notable exception of mother-pup distances. Individually consistent variation in proximity behaviour may be absent as it is so fundamentally important to the survival of the pup that only the variation caused by the physiological system controlling it persists in a population. This physiology-based variation in maternal behaviour would be difficult to remove via natural selection, as this would require drastic mutation of the relevant genes and pathways which produce and utilise oxytocin during reproductive events, such as birth and bonding with a newborn infant. Therefore, when investigating variation in individual behaviour, it is important to distinguish between behaviours which rely solely on cognitive abilities that can show great plasticity in their expression, and behaviours that are tied to hormonal systems which cannot adapt quickly or easily to selection pressures.

## Conclusions

We demonstrate for the first time in a wild species that basal oxytocin concentrations are directly linked to the breeding status of a female and the proximity she maintains to her dependent infant. Quantifying maternal bonds in animals is difficult, typically relying on measuring frequencies of behaviours perceived to correlate with the strength of the mother-offspring bond [79]. We propose that with further validation, measuring basal oxytocin concentrations may represent a novel method of detecting maternal bonds and their strength in non-human species. Oxytocin is a widely conserved hormone that fulfils the same role promoting mother-infant bonds in all mammalian mothers studied to date. Our findings are, therefore, relevant to understanding mother-infant interactions and limitations on infant developmental success across a wide range of species, including humans.

## Supporting Information

S1 Table Behavioural Ethogram. Behavioural ethogram for recording scan data from mother-pup pairs on North Rona in 2010 and 2011.
(DOC)

S2 Table Reported Activity Budgets for Grey Seal Maternal Behaviour. Summary of reported figures for the average percentage time spent in different behaviours by grey seal mothers on a breeding colony, including the behaviour observed post sampling from this study, with the number of scans spent in each behaviour converted into the percentage time spent in each behaviour. Behaviour categories are as defined in Table 1, ethograms of the cited studies were checked for consistency with the ethogram for this study, where appropriate categories from published studies were combined to allow comparison with the categories in Table 1.
(DOC)

S3 Table GAMM outputs for all non-significant models. GAMM outputs for maternal behaviour where plasma oxytocin concentrations did not significantly impact on the dependant variable, their estimates, standard errors and p values.
(DOC)

S4 Table All original data. Behavioural, hormone and individual data
(DOC)

## References

[1] Trillmich F. Parental Care: Adjustments to Conflict and Cooperation In: Kappeler P, editor. Animal Behaviour: Evolution and Mechanisms. Springer-Verlag, Berlin, Germany; 2010 pp. 267–298.

[2] Künkele J, Kenagy GI. Inefficiency of lactation in primiparous rats: the costs of first reproduction. Physiol. Biochem. Zool. 1997; 70: 571–577. 10.1086/515862 9279924

[3] Perry EA, Boness DJ, Fleischer RC. DNA Fingerprinting evidence of nonfilial nursing in grey seals. Mol. Ecol. 1998; 7: 81–85. 9465418 10.1046/j.1365-294x.1998.00313.x

[4] Rahman A, Iqbal Z, Bunn J, Lovel H, Harrington R. Impact of maternal depression on infant nutritional status and illness: a cohort study. Arch. Gen. Psychiat. 2004; 61: 946–952. 15351773 10.1001/archpsyc.61.9.946

[5] Dwyer CM, Lawrence AB. A review of the behavioural and physiological adaptations of hill and lowland breeds of sheep that favour lamb survival. Appl. Anim. Behav. Sci. 2005; 92: 235–260.

[6] Everett-Hincks JM, Lopez-Villalobos N, Blair HT, Stafford KJ. The effect of ewe maternal behaviour score on lamb and litter survival. Livest. Prod. Sci. 2005; 93: 51–61.

[7] Lindström J. Early development and fitness in birds and mammals. Trends Ecol. Evol. 1999; 14: 343–348. 10441307 10.1016/s0169-5347(99)01639-0

[8] Fairbanks LA. Individual differences in maternal style: causes and consequences for mothers and offspring. Adv. Stud. Behav. 1996; 25: 579–611.

[9] Clutton-Brock TH. Reproductive effort and terminal investment in iteroparous animals. Am. Nat. 1984; 123: 212–229.

[10] Conradt L, Clutton-Brock TH, Guinness FE. The relationship between habitat choice and lifetime reproductive success in female red deer. Oecologia 1999; 120: 218–224. 28308082 10.1007/s004420050851

[11] DeGabriel JL, Moore BD, Foley WJ, Johnson CN. The effect of plant defensive chemistry on nutrient availability predict reproductive success in a mammal. Ecology 2009; 90: 711–719. 19341141 10.1890/08-0940.1

[12] Nelson RJ. Chapter 7, Parental Behaviour In: An Introduction to Behavioural Endocrinology. 2nd Edition Sinauer Associates Inc, Massachusetts, USA: 2000 pp 337–394.

[13] Galbally M, Lewis AJ, IJzendoorn MV, Permezel M. The role of oxytocin in mother-infant relations: a systematic review of human studies. Harvard Rev. Psychiat. 2011; 19: 1–14. 10.3109/10673229.2011.549771 21250892

[14] Heinrichs M, Domes G. Neuropeptides and social behaviour: effects of oxytocin and vasopressin in humans. Prog. Brain Res. 2008; 170: 337–350. 10.1016/S0079-6123(08)00428-7 18655894

[15] Insel TR. The challenge of translation in social neuroscience: a review of oxytocin, vasopressin, and affiliative behavior. Neuron 2010; 65: 768–779. 10.1016/j.neuron.2010.03.005 20346754 PMC2847497

[16] Kendrick KM. Oxytocin, motherhood and bonding. Exp. Physiol. 2000; 85: 111–124. 10.1111/j.1469-445x.2000.tb00014.x 10795913

[17] Broad KD, Curley JP, Keverne EB. Mother–infant bonding and the evolution of mammalian social relationships. Philos. T. Roy. Soc. B. 2006; 361: 2199–2214. 10.1098/rstb.2006.1940 PMC1764844 17118933

[18] Feldman R, Weller A, Zagoory-Sharon O, Levine A. Evidence for a neuroendocrinological foundation of human affiliation: plasma oxytocin levels across pregnancy and the postpartum period predict mother-infant bonding. Psychol. Sci. 2007; 18: 965–970. 17958710 10.1111/j.1467-9280.2007.02010.x

[19] Strathearn L, Fonagy P, Amico J, Montague PR. Adult attachment predicts maternal brain and oxytocin response to infant cues. Neuropsychopharmacol. 2009; 34: 2655–2666. 10.1038/npp.2009.103 PMC3041266 19710635

[20] Lévy F, Kendrick KM, Goode JA, Guevara-Guzman R, Keverne EB. Oxytocin and vasopressin release in the olfactory bulb of parturient ewes: changes with maternal experience and effects on acetylcholine, gamma-aminobutyric acid, glutamate and noradrenaline release. Brain Res. 1995; 669: 197–206. 7712175 10.1016/0006-8993(94)01236-b

[21] Da Costa APC, Guevara-Guzman RG, Ohkura S, Goode JA, Kendrick KM. The role of oxytocin release in the paraventricular nucleus in the control of maternal behaviour in the sheep. J. Neuroendocrinol. 1996; 8: 163–177. 8730650 10.1046/j.1365-2826.1996.04411.x

[22] Dwyer CM. Individual variation in the expression of maternal behaviour: a review of the neuroendocrine mechanisms in the sheep. J. Neuroendocrinol. 2008; 20: 526–534. 10.1111/j.1365-2826.2008.01657.x 18266950

[23] Gimpl G, Fahrenholz F. The oxytocin receptor system, structure function and regulation. Physiol. Rev. 2001; 81: 629–683. 11274341 10.1152/physrev.2001.81.2.629

[24] Atzil S, Hendler T, Feldman R. Specifying the neurobiological basis of human attachment: brain, hormones, and behaviour in synchronous and intrusive mothers. Neuropsychopharmacol. 2011; 36: 2603–2615. 10.1038/npp.2011.172 PMC3230500 21881566

[25] Maestripieri D, Hoffman CL, Anderson GM, Carter CS, Higley J.D. Mother-infant interactions in free-ranging rhesus macaques: relationships between physiological and behavioural variables. Physiol. Behav. 2009; 96: 613–619. 10.1016/j.physbeh.2008.12.016 19150451 PMC3955190

[26] Bales KL, van Westerhuyzen JA, Lewis-Reese AD, Grotte ND, Lanter JA, Carter CS. Oxytocin has dose-dependent developmental effects on pair-bonding and alloparental care in female prairie voles. Horm. Behav. 2007; 52: 274–279. 17553502 10.1016/j.yhbeh.2007.05.004 PMC1978481

[27] Madden JR, Clutton-Brock TH. Experimental peripheral administration of oxytocin elevates a suite of cooperative behaviours in a wild social mammal. P. Roy. Soc. B. 2011; 278: 1189–1194. 10.1098/rspb.2010.1675 PMC3049071 20926437

[28] Olsson IAS, Nevison CM, Patterson-Kane EG, Sherwin CM, Van de Weerd HA, Wübel H. Understanding behaviour: the relevance of ethological approaches in laboratory animal science. Appl. Anim. Behav. Sci. 2003; 81: 245–264.

[29] Newberry RC. Environmental enrichment: increasing the biological relevance of captive environments. Appl. Anim. Behav. Sci.

[30] Robinson KJ, Hazon N, Lonergan M, Pomeroy PP. Validation of an enzyme-linked immunoassay (ELISA) plasma oxytocin in a novel mammal species reveals potential errors induced by sampling procedure. J. Neurosci. Meth. 2014; 226: 73–39.2005; 44: 229–243. 10.1016/j.jneumeth.2014.01.019 24485867

[31] Pomeroy PP, Fedak MA, Rothery P, Anderson S. Consequences of maternal size for reproductive expenditure and pupping success of grey seals at North Rona, Scotland. J. Anim. Ecol. 1999; 68: 235–253.

[32] Hiby L, Lovell P. Computer aided matching of natural markings: a prototype system for grey seals. Reports of the International Whaling Commission, Special. 1990; 12: 57–61.

[33] Iverson SJ, Bowen WD, Boness DJ, Oftedal OT. The effect of maternal size and milk energy output on pup growth in grey seals (*Halichoerus grypus*). Physiol. Zool. 1993; 66: 61–88.

[34] Hall AJ, McConnell BJ, Barker RJ. Factors affecting first-year survival in grey seals and their implications for life history strategy. J. Anim. Ecol. 2001; 70: 138–149.

[35] Fogden SCL. Mother-young behaviour at grey seal breeding beaches. J. Zool. 1971; 164: 61–92.

[36] McCulloch S, Pomeroy PP, Slater PJB. Individually distinctive pup vocalizations fail to prevent allo-suckling in grey seals. Can. J. Zool. 1999; 77: 716–723.

[37] Poland VF, Pomeroy PP, Twiss SD, Graves JA. Fine‐scale study finds limited evidence of kin clustering in a gray seal colony. Mar. Mammal Sci. 2008; 24: 371–387.

[38] Feldman R, Gordon I, Schneiderman I, Weisman O, Zagoory-Sharon O. Natural variations in maternal and paternal care are associated with systematic changes in oxytocin following parent-infant contact. Psychoneuroendocrinol. 2010; 35: 113–1141. 10.1016/j.psyneuen.2010.01.013 20153585

[39] Holt-Lundstad J, Birmingham W, Light KC. The influence of depressive symptomatology and perceived stress on plasma and salivary oxytocin before, during and after a support enhancement intervention. Psychoneuroendocrinol. 2011; 36: 1249–1256. 10.1016/j.psyneuen.2011.03.007 21507578

[40] Modahl C, Green LA, Fein D, Morris M, Waterhouse L, Feinstein C, et al Plasma oxytocin levels in autistic children. Biol. Psychiat. 1998; 43: 270–277. 9513736 10.1016/s0006-3223(97)00439-3

[41] Scantamburlo G, Hansenne M, Fuchs S, Pitchot W, Marechal P, Pequeux C, et al Plasma oxytocin levels and anxiety in patients with major depression. Psychoneuroendocrinol. 2007; 32: 407–410. 10.1016/j.psyneuen.2007.01.009 17383107

[42] El-Masry N, Soliman A, Moety HA. Alterations of prolyl endopeptidase, oxytocin and vasopressin activity in the plasma of autistic children. Current Psychiatry 2010; 17: 31–37.

[43] Hoge EA, Lawson EA, Metcalf CA, Keshaviah A, Zak PJ, Pollack MH, et al Plasma oxytocin immunoreactive products and response to trust in patients with social anxiety disorder. Depress. Anxiety 2012; 29: 924–930. 10.1002/da.21973 22807189 PMC3751166

[44] Levine A, Zagoory-Sharon O, Feldman R, Weller, A. Oxytocin during pregnancy and early postpartum: Individual patterns and maternal-fetal attachment. Peptides 2007; 28: 1162–1169. 17513013 10.1016/j.peptides.2007.04.016

[45] Meyer-Lindenberg A, Domes G, Kirsch P, Heinrichs M. Oxytocin and vasopressin in the human brain: social neuropeptides for translational medicine. Nat. Rev. Neurosci. 2011; 12: 524–538. 10.1038/nrn3044 21852800

[46] Neumann ID, Landgraf R. Balance of brain oxytocin and vasopressin: implications for anxiety, depression, and social behaviours. Trends Neurosci. 2012; 35: 649–659. 10.1016/j.tins.2012.08.004 22974560

[47] Smout S, King R, Pomeroy P. Estimating demographic parameters for capture–recapture data in the presence of multiple mark types. Environ. Ecol. Stat. 2011; 18: 331–347.

[48] Kovacs KM. Maternal behaviour and early behavioural ontogeny of grey seals (Halichoerus grypus) on the Isle of May, UK. J. Zool. 1987; 213: 697–715.

[49] Rydén G, Sjöholm I. Half-life of oxytocin in blood of pregnant and non-pregnant women. Acta Endocrinol-Cop. 1969; 61: 425–431. 5820054

[50] Haaland P, Samarov D, McVey E. calibFit: Statistical models and tools for assay calibration. R package version 2.1.0/r17. 2011. http://R-Forge.R-project.org/projects/calibfun/

[51] R Core Development Team. R: A language and environment for statistical computing R Foundation for Statistics Computing, Vienna, Austria 2012 http://www.R-project.org.

[52] Haller MA, Kovacs KM, Hammill MO. Maternal behaviour and energy investment by grey seals (*Halichoerus grypus)* breeding on land-fast ice. Can. J. Zool. 1996; 74: 1531–1541.

[53] Redman P. The role of temporal, spatial and kin associations in grey seal breeding colonies. PhD thesis, The University of St Andrews, Scotland. 2002.

[54] Culloch RM. The application of modern statistical approaches to identify consistent individual differences in the behaviour of wild postpartum female grey seals (Halichorus grypus). PhD thesis, Durham University, UK. 2012.

[55] Twiss SD, Culloch R, Pomeroy PP. An in-field experimental test of pinniped behavioural types. Mar. Mammal Sci. 2012; 28: 280–294.

[56] Twiss SD, Cairns C, Culloch RM, Richards SA, Pomeroy PP. 2012b Variation in female grey seal (*Halichoerus grypus)* reproductive performance correlates to proactive-reactive behavioural types. PLOS one. 7, e49598. 23166723 10.1371/journal.pone.0049598 PMC3500302

[57] Wood S. Generalized Additive Models: An introduction with R. Chapman and Hall/CRC 2006.

[58] Fedak MA, Anderson SS. The energetics of lactation: accurate measurements from a large wild mammal, the grey seal (*Halichoerus grypus*). J. Zool. 1982; 198: 473–479.

[59] Boness DJ, Bowen WD. The evolution of maternal care in pinnipeds. Bioscience 1996; 46: 645–654.

[60] Wood S. mgcv: Mixed GAM Computation Vehicle with GCV/AIC/REML smoothness estimation. 2012. http://cran.r-project.org/web/packages/mgcv/index.html

[61] Wood S. Low-rank scale-invariant tensor product smooths for generalized additive mixed models. Biometrics 2006; 62: 1025–1036. 17156276 10.1111/j.1541-0420.2006.00574.x

[62] Wood S. Fast stable restricted maximum likelihood and marginal likelihood estimation of semiparametric generalized linear models. J. Roy. Stat. Soc. 2011; 73: 3–36.

[63] Szeto A, McCabe P, Nation DA, Tabak BA, Rossetti MA, McCullough ME, et al Evalutaion of enzyme immunoassay and radioimmunoassay methods for the measurement of plasma oxytocin. Psychosom. Med. 2011; 73: 393–400. 10.1097/PSY.0b013e31821df0c2 21636661 PMC3118424

[64] Gossen A, Hahn A, Westphal L, Prinz S, Schultz RT, Gründer G, et al Oxytocin plasma concentrations after single intranasal oxytocin administration–a study in healthy men. Neuropeptides 2012; 46: 211–215. 10.1016/j.npep.2012.07.001 22884888

[65] Dawood MY, Khan-Dawood S, Wahi RS, Fuchs F. Oxytocin release and plasma anterior pituitary and gonadal hormones in women during lactation. J. Clin. Endocr. Metab. 1981; 52: 678–683. 6782115 10.1210/jcem-52-4-678

[66] Drewett RF, Bowen-Jones A, Dogterom J. Oxytocin levels during breast-feeding in established lactation. Horm. Behav. 1982; 16: 245–248. 7118090 10.1016/0018-506x(82)90023-x

[67] Higuchi T, Honda K, Fukuoka T, Negoro H, Wakabayashi K. Release of oxytocin during suckling and parturition in the rat. J. Endocrinol. 1985; 105: 339–346. 3998651 10.1677/joe.0.1050339

[68] Skrundz M, Bolten M, Nast I, Hellhammer DH, Meinlschmidt G. Plasma oxytocin concentration during pregnancy is associated with development of postpartum depression. Neuropsychopharmacol. 2011; 36: 1886–1893. 10.1038/npp.2011.74 PMC3154107 21562482

[69] Alexander G, Poindron P, Le Neindre P, Stevens D, Lévy F, Bradley L. Importance of the first hour post-partum for exclusive maternal bonding in sheep. Appl. Anim. Behav. Sci. 1986; 16: 295–300.

[70] Burton RW, Anderson SS, Summers CF. Perinatal activities in the grey seal (*Halichoerus grypus*). J. Zool. 1975; 177: 197–201.

[71] Turner RA, Altemus M, Enos T, Cooper B, McGuinness T. Preliminary research on plasma oxytocin in normal cycling women: investigating emotion and interpersonal distress. Psychiatr. 1999; 62: 97–113. 10.1080/00332747.1999.11024859 10420425

[72] Landgraf R, Neumann ID. Vasopressin and oxytocin release within the brain: a dynamic concept of multiple and variable modes of neuropeptide communication. Front. Neuroendocrin. 2004; 25: 150–176. 10.1016/j.yfrne.2004.05.001 15589267

[73] Boyd JM, Campbell RN. The grey seal (Halichoerus grypus) at North Rona, 1959 to 1968. J. Zool. 1971; 164: 469–512.

[74] Anderson SS, Baker JR, Prime JH, Baird A. Mortality in grey seal pups: incidence and causes. J. Zool. 1979; 189: 407–417.

[75] Baker JR, Baker R. Effects of environment on grey seal (*Halichoerus grypus*) pup mortality. Studies on the Isle of May. J. Zool. 1988; 216: 529–537.

[76] Baskerville TA, Douglas AJ. Dopamine and oxytocin interactions underlying behaviours: potential contributions to behavioural disorders. CNS Neurosci. Ther. 2010; 16: 92–123. 10.1111/j.1755-5949.2010.00154.x PMC6493805 20557568

[77] Tsuneoka Y, Maruyama T, Yoshida S, Nishimori K, Kato T, Numan M, et al Functional, anatomical and neurochemical differentiation of medial preoptic area subregions in relation to maternal behaviour in the mouse. J Comp. Neurol. 2013; 521: 1633–1663. 10.1002/cne.23251 23124836

[78] Otero-García M, Agustín-Pavón C, Lanuza E, Martínez-García F. Distribution of oxytocin and co-localization with arginine vasopressin in the brain of mice. 2015; Brain Struct. Funct. 1–29. 26388166 10.1007/s00429-015-1111-y

[79] Houpt KA. Formation and dissolution of the mare-foal bond. Appl. Anim. Behav. Sci. 2002; 78: 319–328.

