# Supplementary material for: Maternal Oxytocin Is Linked to Close Mother-Infant Proximity in Grey Seals (Halichoerus grypus)
Source: PLoS One. 2015 Dec 23;10(12):e0144577. doi: 10.1371/journal.pone.0144577 (PMC4689390; doi:10.1371/journal.pone.0144577)
Supplement: S1 Table — Behavioural ethogram for recording scan data from mother-pup pairs on North Rona in 2010 and 2011. (DOC) [file pone.0144577.s001.doc]

**S1 Table. Behavioural Ethogram.** Behavioural ethogram for recording scan data from mother-pup pairs on North Rona in 2010 and 2011

| **Behaviour type** | **Specific behaviour categories** | **Definition.** |
| --- | --- | --- |
| **General behaviour** | Rest | Individual stationary, with head down on the substrate. |
|  | Head up rest | Individual stationary, with head up but neck not extended or looking specifically at surroundings or another seal/animal. |
|  | Alert | Head up, neck extended, looking around or directly at something excluding the dependant pup. |
|  | Locomotion | Travelling more than two body lengths from initial position. |
|  | Comfort move | Scratching or rubbing movements. |
|  | Out of sight | Focal mother not in sight of the observer. |
|  | Other | Any behaviours that are not specified by the ethogram. |
| **Maternal**  **Aggressive behaviour** | Open mouth threat (female) | Mouth open and facing towards another female grey seal, typically with head held low and neck extended. |
|  | Open mouth threat (male) | As above but directed towards a male grey seal. |
|  | Aggressive flippering | One or both fore flippers brandished rapidly at another seal, may or may not come into physical contact. |
|  | Lunge | Bite attempt, neck extends rapidly and then retracts without contact with the subject. |
|  | Bite | Actual physical contact of one animal’s open mouth to any part of the other subject’s body, in a rapid and aggressive manner. |
|  | Chase | Rapid locomotion after an individual seal, covering a distance of more than two body lengths. |
|  | Flee | Rapid locomotion away from another seal, resulting in separation of more than one body length between the two individuals. |
|  | Vocalisation | Any noise produced by the focal animal, note that recording vocalisations is potentially confounded by wind and distance from the hide. |
| **Maternal behaviour** | Flippering | One fore flipper gently rubbing the surface of the pup’s head or body to encourage nursing. |
|  | Presenting | Mother rolls onto her side to expose nipples to allow pup to nurse. |
|  | Nursing | Pup is in contact with the mother’s nipple. |
|  | Check pup | Head up or neck extended, looking directly at the dependant pup. |
|  | Interact with pup | Any form of a mother physically touching her pup that does not fall into the above categories, with or without the pup reciprocating. |
|  | Birth | Mother gives birth to a live or dead pup. |
| **Sexual behaviour** | Copulation (attempted) | Interaction between the mother and a male seal in which the male is attempting to copulate, but is unsuccessful due to the mother attempting to drive the male away via aggressive behaviours. |
|  | Copulation (failed) | Copulation attempt where the focal mother did not attempt to drive the male away but the copulation was still unsuccessful. |
|  | Copulation (successful) | Male successfully copulates with the focal mother |
