# Supplementary material for: Maternal Oxytocin Is Linked to Close Mother-Infant Proximity in Grey Seals (Halichoerus grypus)
Source: PLoS One. 2015 Dec 23;10(12):e0144577. doi: 10.1371/journal.pone.0144577 (PMC4689390; doi:10.1371/journal.pone.0144577)
Supplement: S2 Table — Summary of reported figures for the average percentage time spent in different behaviours by grey seal mothers on a breeding colony, including the behaviour observed post sampling from this study, with the number of scans spent in each behaviour converted into the percentage time spent in each behaviour. Behaviour categories are as defined in Table 1, ethograms of the cited studies were checked for consistency with the ethogram for this study, where appropriate categories from published studies were combined to allow comparison with the categories in Table 1. (DOC) [file pone.0144577.s002.doc]

**S2 Table. Reported Activity Budgets for Grey Seal Maternal Behaviour.** Summary of reported figures for the average percentage time spent in different behaviours by grey seal mothers on a breeding colony, including the behaviour observed post sampling from this study (Robinson *et al* 2015), with the number of scans spent in each behaviour converted into the percentage time spent in each behaviour. Behaviour categories are as defined in Table 1, ethograms of the cited studies were checked for consistency with the ethogram for this study, where appropriate categories from published studies were combined to allow comparison with the categories in Table 1.

| **Study** | Anderson and Harwood 1985 | Anderson and Harwood 1985 | Haller *et al.* 1996 | Haller *et al.* 1996 | Twiss *et al.* 2000 | Twiss *et al.* 2000 | Twiss *et al.* 2012 | Culloch 2012 | Robinson *et al.* 2015 |
| --- | --- | --- | --- | --- | --- | --- | --- | --- | --- |
| **Mean/median** | Mean | Mean | Mean | Mean | Median | Median | Mean | Median | Mean |
| **Sampling Regime** | Scans | Scans | Scans | Scans | Scans | Scans | Scans | Scans | Scans |
| **Study Site** | North Rona, Scotland | Monach Isles, Scotland | Amet Island, Canada | Gulf of St Lawrence, Canada | West Rona beach, Isle of May, Scotland | Tarbet, Isle of May, Scotland | North Rona, Scotland | North Rona, Scotland | North Rona, Scotland |
| **Behaviour (%)** |  |  |  |  |  |  |  |  |  |
| Resting | 79.7 | 71.0 | 86.2 | 89.5 | 79.09 | 77.1 | 79.1 | 79.8 | 80.9 |
| Nursing | 1.8 | 3.6 | 8.4 | 6.1 | na^1^ | na^1^ | 10.2 | 3.8 | 5.04 |
| Interacting with pup | 1.2 | 1.1 | 0.7 | 0.4 | 5.2 | 4.6 | 1.7 | 1.28 | 1.3 |
| Checking pup | na^2^ | na^2^ | 3.0 | 2.2 | na^2^ | na^2^ | 2.5 | 1.9 | 3.5 |
| Alert | 10.2 | 15.0 | 11.2 | 13.6 | 10.4 | 11.4 | 6.5 | 6.04 | 3.6 |
| Aggression | 1.0 | 1.4 | 2.0 | 1.4 | 1.7 | 1.8 | 0.7 | 0.7 | 0.7 |
| Locomotion | 1.3 | 4.6 | 2.4 | 1.0 | 0 | 1.9 | 1.4 | 0.7 | 1.2 |

na^1^ Nursing behaviours included in the Interacting with pup behaviours

na^2^ Checking pup behaviour included in the Alert behaviour

References: **Anderson**, S. S., & Harwood, J. (1985). Time budgets and topography: how energy reserves and terrain determine the breeding behaviour of grey seals. *Animal Behaviour*, *33*(4), 1343-1348. **Haller**, M.A., Kovacs, K.M. and Hammill, M.O. (1996) Maternal behaviour and energy investment by grey seals (*Halichoerus grypus)* breeding on land-fast ice.  *Canadian Journal of Zoology vol 74, P1531-1541*. **Twiss**, S. D., Caudron, A., Pomeroy, P. P., Thomas, C. J., & Mills, J. P. (2000). Finescale topographical correlates of behavioural investment in offspring by female grey seals, Halichoerus grypus. *Animal behaviour*, *59*(2), 327-33. **Twiss**, S.D., Cairns, C., Culloch, R.M., Richards, S.A. and Pomeroy, P.P (2012) Variation in female grey seal (*Halichoerus grypus)* reproductive performance correlates to proactive-reactive behavioural types. *PLOS one vol 7, No. 11, e49598.* **Culloch**, R.M. (2012) The application of modern statistical approaches to identify consistent individual differences in the behaviour of wild postpartum female grey seals (*Halichorus grypus*). *PhD thesis, Durham University, UK.* **Robinson** *et al.* 2015 refers to data from this manuscript.
