# Supplementary material for: Maternal Oxytocin Is Linked to Close Mother-Infant Proximity in Grey Seals (Halichoerus grypus)
Source: PLoS One. 2015 Dec 23;10(12):e0144577. doi: 10.1371/journal.pone.0144577 (PMC4689390; doi:10.1371/journal.pone.0144577)
Supplement: S3 Table — GAMM outputs for maternal behaviour where plasma oxytocin concentrations did not significantly impact on the dependant variable, their estimates, standard errors and p values. (DOC) [file pone.0144577.s003.doc]

**S3 Table. GAMM outputs for all non-significant models.** GAMM outputs for maternal behaviour where plasma oxytocin concentrations did not significantly impact on the dependant variable, their estimates, standard errors and p values.

| **Dependant variable** | **Explanatory variable** | **Estimate** | **Standard Error** | **P value** |
| --- | --- | --- | --- | --- |
| **Resting** | Maternal plasma oxytocin concentration (pg/ml) | -0.0036 | 0.0068 | 0.59 |
|  | Sample timing during the season (early/late) | 0.1 | 0.041 | 0.011 |
|  | Pup sex (male/female) | -1.11 | 0.17 | <0.001 |
|  | Pup birth date | -0.15 | 0.025 | <0.001 |
|  | Number of days behaviour was recorded after sampling | -0.065 | 0.017 | <0.001 |
|  | Smooth term for mother’s identity | Na | Na | <0.001 |
| **Nursing** | Maternal plasma oxytocin concentration (pg/ml) | 0.05 | 0.012 | 0.21 |
|  | Sample timing during the season (early/late) | -0.33 | 0.074 | <0.001 |
|  | Pup sex (male/female) | 0.81 | 0.27 | 0.0029 |
|  | Pup birth date | 0.18 | 0.045 | <0.001 |
|  | Number of days behaviour was recorded after sampling | 0.12 | 0.031 | <0.001 |
|  | Smooth term for mother’s identity | Na | Na | <0.001 |
| **Alert** | Maternal plasma oxytocin concentration (pg/ml) | 0.0076 | 0.014 | 0.57 |
|  | Sample timing during the season (early/late) | -0.0056 | 0.085 | 0.95 |
|  | Pup sex (male/female) | 0.51 | 0.22 | 0.019 |
|  | Pup birth date | 0.022 | 0.028 | 0.43 |
|  | Number of days behaviour was recorded after sampling | -0.063 | 0.033 | 0.056 |
|  | Smooth term for mother’s identity | Na | Na | <0.001 |
| **Locomotion** | Maternal plasma oxytocin concentration (pg/ml) | 0.016 | 0.023 | 0.48 |
|  | Sample timing during the season (early/late) | 0.85 | 0.15 | <0.001 |
|  | Pup sex (male/female) | 0.61 | 0.22 | 0.0058 |
|  | Pup birth date | 0.016 | 0.024 | 0.52 |
|  | Number of days behaviour was recorded after sampling | 0.049 | 0.05 | 0.33 |
|  | Smooth term for mother’s identity | Na | Na | <0.001 |
| **Interacting with pup** | Maternal plasma oxytocin concentration (pg/ml) | 0.018 | 0.031 | 0.56 |
|  | Sample timing during the season (early/late) | -0.27 | 0.15 | 0.059 |
|  | Pup sex (male/female) | 1.2 | 0.67 | 0.069 |
|  | Pup birth date | 0.44 | 0.086 | <0.001 |
|  | Number of days behaviour was recorded after sampling | 0.37 | 0.072 | <0.001 |
|  | Smooth term for mother’s identity | Na | Na | <0.001 |
| **Check pup** | Maternal plasma oxytocin concentration (pg/ml) | -0.025 | 0.014 | 0.068 |
|  | Number of days behaviour was recorded after sampling | 0.14 | 0.03 | <0.001 |
|  | Smooth term for mother’s identity | Na | Na | <0.001 |
