# Supplementary material for: Maternal Oxytocin Is Linked to Close Mother-Infant Proximity in Grey Seals (Halichoerus grypus)
Source: PLoS One. 2015 Dec 23;10(12):e0144577. doi: 10.1371/journal.pone.0144577 (PMC4689390; doi:10.1371/journal.pone.0144577)
Supplement: S4 Table — Behavioural, hormone and individual data (DOC) [file pone.0144577.s004.doc]

**S4 Table. Original Data.** Behavioural, hormone and individual data

|  | | | | | | | | | Behavioural Scan Data | | | | | | | |
| --- | --- | --- | --- | --- | --- | --- | --- | --- | --- | --- | --- | --- | --- | --- | --- | --- |
| Breeding status | Seal ID | Sample date | Observation date | Sample Timing | Day of pup birth within the breeding season | Pup sex | Plasma oxytocin (pg/ml) | Mean distance from pup (seal body lengths) | Resting | Nursing | Alert | Locomotion | Aggression | Interaction with pup | Checking pup | Other behaviours |
| Mother | 50345 | 05.10.10 | 06.10.10 | early | 3 | M | 10.9 | 0.26 | 776 | 25 | 15 | 16 | 7 | 1 | 31 | 4 |
| Mother | 52089 | 05.10.10 | 06.10.10 | early | 4 | F | 6.1 | 2.25 | 700 | 24 | 68 | 21 | 1 | 1 | 39 | 26 |
| Mother | 52089 | 15.10.10 | 16.10.10 | late | 4 | F | 4.5 | 7.86 | 669 | 11 | 71 | 21 | 13 | 2 | 31 | 17 |
| Mother | ‘Discus' | 05.10.10 | 07.10.10 | early | 3 | M | 8.5 | 1.27 | 693 | 16 | 56 | 11 | 0 | 2 | 62 | 21 |
| Mother | 54422 | 05.10.10 | 08.10.10 | early | 3 | F | 6.3 | 0.81 | 699 | 66 | 23 | 7 | 1 | 17 | 19 | 26 |
| Mother | 54422 | 15.10.10 | 18.10.10 | late | 3 | F | 4.6 | 0.31 | 581 | 49 | 24 | 8 | 4 | 24 | 26 | 55 |
| Mother | 54203/5 | 05.10.10 | 08.10.10 | early | 5 | M | 8.2 | 4.44 | 728 | 49 | 16 | 8 | 9 | 6 | 16 | 26 |
| Mother | 51241/2 | 09.10.10 | 14.10.10 | early | 8 | F | 7.3 | 1.02 | 656 | 29 | 53 | 15 | 3 | 1 | 21 | 36 |
| Mother | 51241/2 | 19.10.10 | 21.10.10 | late | 8 | F | 6.9 | 2.78 | 665 | 50 | 27 | 6 | 6 | 0 | 33 | 21 |
| Mother | 15.4 | 09.10.10 | 10.10.10 | early | 9 | M | 7.8 | 0.67 | 683 | 45 | 14 | 9 | 5 | 24 | 36 | 42 |
| Mother | 15.4 | 19.10.10 | 20.10.10 | late | 9 | M | 8.6 | 0.55 | 674 | 40 | 16 | 6 | 2 | 12 | 30 | 12 |
| Mother | 52219 | 09.10.10 | 13.10.10 | early | 8 | F | 7.5 | 4.26 | 487 | 24 | 35 | 20 | 6 | 3 | 15 | 26 |
| Mother | 52219 | 22.10.10 | 23.10.10 | late | 8 | F | 5.9 | 0.31 | 544 | 78 | 43 | 6 | 2 | 22 | 66 | 20 |
| Mother | ‘Sigma' | 15.10.10 | 21.10.10 | early | 14 | F | 6.7 | 1.44 | 688 | 48 | 19 | 1 | 2 | 0 | 26 | 24 |
| Mother | ‘Sigma' | 26.10.10 | 28.10.10 | late | 14 | F | 8.4 | 1.03 | 581 | 55 | 33 | 4 | 4 | 45 | 29 | 31 |
| Mother | 52232 | 12.10.10 | 16.10.10 | early | 9 | M | 7.4 | 16.61 | 670 | 21 | 43 | 26 | 21 | 0 | 11 | 21 |
| Mother | 52232 | 22.10.10 | 24.10.10 | late | 9 | M | 9.1 | 0.26 | 619 | 97 | 19 | 4 | 4 | 6 | 12 | 25 |
| Mother | 50873/4 | 19.10.10 | 20.10.10 | early | 18 | F | 8.3 | 2.63 | 609 | 60 | 32 | 26 | 5 | 1 | 35 | 24 |
| Mother | 50873/4 | 31.10.10 | 01.11.10 | late | 18 | F | 8.8 | 0.19 | 614 | 44 | 23 | 1 | 2 | 10 | 16 | 16 |
| Mother | ‘Splits' | 22.10.10 | 25.10.10 | early | 20 | M | 7.5 | 0.34 | 671 | 54 | 31 | 6 | 3 | 25 | 32 | 14 |
| Mother | ‘Splits' | 31.10.10 | 01.11.10 | late | 20 | M | 6.7 | 0.82 | 622 | 25 | 15 | 2 | 2 | 24 | 21 | 15 |
| Mother | 51253/4 | 07.10.11 | 08.10.11 | early | 6 | M | 24.9 | 0.4 | 680 | 33 | 23 | 6 | 4 | 4 | 28 | 14 |
| Mother | 51253/4 | 20.10.11 | 21.10.11 | late | 6 | M | 6.6 | 0.4 | 448 | 28 | 18 | 1 | 15 | 2 | 11 | 5 |
| Mother | 54422 | 07.10.11 | 08.10.11 | early | 6 | F | 7.6 | 0.4 | 717 | 16 | 20 | 2 | 1 | 2 | 26 | 8 |
| Mother | 54422 | 17.10.11 | 21.10.11 | late | 6 | F | 12.2 | 0.3 | 427 | 26 | 19 | 1 | 0 | 2 | 15 | 38 |
|  | | | | | | | | | Behavioural Scan Data | | | | | | | |
| Breeding status | Seal ID | Sample date | Observation date | Sample Timing | Day of pup birth within the breeding season | Pup sex | Plasma oxytocin (pg/ml) | Mean distance from pup (seal body lengths) | Resting | Nursing | Alert | Locomotion | Maternal  Aggression | Interaction with pup | Checking pup | Other behaviours |
| Mother | 50866/7 | 17.10.11 | 22.10.11 | late | 3 | F | 8.3 | 0.8 | 563 | 63 | 32 | 8 | 17 | 14 | 54 | 41 |
| Mother | 51241/2 | 09.10.11 | 15.10.11 | early | 9 | F | 13.2 | 1.3 | 693 | 1 | 33 | 16 | 3 | 0 | 29 | 17 |
| Mother | 51241/2 | 24.10.11 | 26.10.11 | late | 9 | F | 8.9 | 0.5 | 573 | 100 | 26 | 8 | 6 | 2 | 28 | 49 |
| Mother | ‘Jolene' | 12.10.11 | 13.10.11 | early | 10 | M | 15.6 | 0.7 | 708 | 11 | 28 | 14 | 31 | 14 | 23 | 7 |
| Mother | ‘Jolene' | 24.10.11 | 25.10.11 | late | 10 | M | 8.7 | 0.4 | 626 | 36 | 34 | 4 | 3 | 19 | 18 | 52 |
| Mother | 15.4 | 14.10.11 | 15.10.11 | early | 7 | M | 13.7 | 0.6 | 573 | 33 | 20 | 4 | 9 | 19 | 27 | 19 |
| Mother | 15.4 | 24.10.11 | 25.10.11 | late | 7 | M | 9.9 | 0.8 | 588 | 55 | 20 | 8 | 3 | 32 | 46 | 40 |
| Mother | O8 | 14.10.11 | 16.10.11 | early | 12 | F | 3.3 | 13 | 639 | 0 | 12 | 26 | 1 | 0 | 1 | 109 |
| Mother | 50873/4 | 19.10.11 | 22.10.11 | early | 14 | M | 7.4 | 1.4 | 708 | 35 | 17 | 7 | 0 | 4 | 13 | 8 |
| Mother | ‘Sigma' | 21.10.11 | 22.10.11 | early | 15 | F | 9.7 | 3.9 | 628 | 28 | 18 | 16 | 0 | 26 | 52 | 24 |
| Mother | 50345 | 15.10.10 | na | late | na | na | 7.8 | na | na | na | na | na | na | na | na | na |
| Mother | ‘Monach L’ | 05.10.10 | na | early | na | na | 14.3 | na | na | na | na | na | na | na | na | na |
| Mother | ‘Monach L’ | 15.10.10 | na | late | na | na | 8.5 | na | na | na | na | na | na | na | na | na |
| Mother | 51253/4 | 05.10.10 | na | early | na | na | 5.4 | na | na | na | na | na | na | na | na | na |
| Mother | 51253/4 | 19.10.10 | na | late | na | na | 5.9 | na | na | na | na | na | na | na | na | na |
| Mother | ‘Discus’ | 15.10.10 | na | late | na | na | 6.4 | na | na | na | na | na | na | na | na | na |
| Mother | 54203/5 | 15.10.10 | na | late | na | na | 3.5 | na | na | na | na | na | na | na | na | na |
| Mother | 51245/6 | 09.10.10 | na | early | na | na | 4.2 | na | na | na | na | na | na | na | na | na |
| Mother | 51245/6 | 19.10.10 | na | late | na | na | 3.6 | na | na | na | na | na | na | na | na | na |
| Mother | ‘Jolene’ | 09.10.10 | na | early | na | na | 4.5 | na | na | na | na | na | na | na | na | na |
| Mother | ‘Jolene’ | 22.10.10 | na | late | na | na | 4.6 | na | na | na | na | na | na | na | na | na |
| Mother | O8 | 09.10.10 | na | early | na | na | 9.9 | na | na | na | na | na | na | na | na | na |
| Mother | O8 | 19.10.10 | na | late | na | na | 5.2 | na | na | na | na | na | na | na | na | na |
| Mother | 51057 | 26.10.10 | na | early | na | na | 15.8 | na | na | na | na | na | na | na | na | na |
| Mother | 51057 | 31.10.10 | na | late | na | na | 3.8 | na | na | na | na | na | na | na | na | na |
| Mother | 52271/2 | 02.10.11 | na | early | na | na | 10.6 | na | na | na | na | na | na | na | na | na |
| Mother | 52271/2 | 12.10.11 | na | late | na | na | 9.7 | na | na | na | na | na | na | na | na | na |
|  | | | | | | | | | Behavioural Scan Data | | | | | | | |
| Breeding status | Seal ID | Sample date | Observation date | Sample Timing | Day of pup birth within the breeding season | Pup sex | Plasma oxytocin (pg/ml) | Mean distance from pup (seal body lengths) | Resting | Nursing | Alert | Locomotion | Maternal  Aggression | Interaction with pup | Checking pup | Other behaviours |
| Mother | 50866/7 | 07.10.11 | na | early | na | na | 8.2 | na | na | na | na | na | na | na | na | na |
| Mother | ‘M8-2011’ | 07.10.11 | na | early | na | na | 4.3 | na | na | na | na | na | na | na | na | na |
| Mother | ‘M8-2011’ | 17.10.11 | na | late | na | na | 6.4 | na | na | na | na | na | na | na | na | na |
| Mother | 51245/6 | 07.10.11 | na | early | na | na | 6.9 | na | na | na | na | na | na | na | na | na |
| Mother | 51245/6 | 17.10.11 | na | late | na | na | 8.9 | na | na | na | na | na | na | na | na | na |
| Mother | O8 | 27.10.11 | na | late | na | na | 6.3 | na | na | na | na | na | na | na | na | na |
| Mother | 50873/4 | 30.10.11 | na | late | na | na | 13 | na | na | na | na | na | na | na | na | na |
| Mother | 51057 | 20.10.11 | na | early | na | na | 6.3 | na | na | na | na | na | na | na | na | na |
| Mother | 51057 | 30.10.11 | na | late | na | na | 5.6 | na | na | na | na | na | na | na | na | na |
| Mother | ‘Sigma’ | 30.10.11 | na | late | na | na | 8.3 | na | na | na | na | na | na | na | na | na |
| Non-breeding | 73002/3 | 15.11.11 | na | na | na | na | 4.0 | na | na | na | na | na | na | na | na | na |
| Non-breeding | 73004/5 | 15.11.11 | na | na | na | na | 4.7 | na | na | na | na | na | na | na | na | na |
| Non-breeding | 73012/3 | 18.11.11 | na | na | na | na | 3.8 | na | na | na | na | na | na | na | na | na |
| Non-breeding | 73072/3 | 27.11.11 | na | na | na | na | 4.9 | na | na | na | na | na | na | na | na | na |
| Non-breeding | 73092/3 | 28.11.11 | na | na | na | na | 8.3 | na | na | na | na | na | na | na | na | na |
| Non-breeding | 73096/7 | 29.11.11 | na | na | na | na | 5.9 | na | na | na | na | na | na | na | na | na |
| Non-breeding | 73194/5 | 01.12.11 | na | na | na | na | 1.7 | na | na | na | na | na | na | na | na | na |
| Non-breeding | 73198/9 | 02.12.11 | na | na | na | na | 4.1 | na | na | na | na | na | na | na | na | na |
